# Growing older with HIV in the Treat‐All Era

**DOI:** 10.1002/jia2.25997

**Published:** 2022-09-29

**Authors:** Reena Rajasuriar, Heidi M. Crane, Aggrey S. Semeere

**Affiliations:** ^1^ Faculty of Medicine University of Malaya Kuala Lampur Malaysia; ^2^ University of Washington Seattle Washington USA; ^3^ Research Department Infectious Diseases Institute Makerere University Kampala Uganda

**Keywords:** HIV, ageing, comorbidity, lifespan, healthspan, treat‐all

One of the most impactful global public health interventions of modern times has been antiretroviral therapy (ART). This has led to reduced HIV transmission; reduced morbidity from opportunistic infections; and dramatically reduced mortality [1, 2, 3], resulting in many people with HIV (PWH) surviving into middle and old age. However, even as the lifespan of PWH has increased and begun to more closely approach those without HIV, PWH continue to experience high rates of comorbidities and functional decline, with many comorbidities occurring at higher rates and/or at younger ages than those without HIV. In addition, geriatric syndromes, such as frailty and falls, are becoming more prevalent in PWH. Thus, there is an urgent need to focus on the healthspan of PWH rather than just mortality.

Healthspan, in contrast to lifespan, is defined as the time someone is healthy, not just alive [4]. As the population of older PWH increases, more studies are needed that address the myriad of unprecedented and unique challenges that ensue. This supplement presents a range of studies utilizing varied methodologies examining questions along the spectrum of growing older while living with HIV, ranging from resilience and suicidal risk among young adult PWH to comorbidities, cognition and mental health disorders among older PWH, including unmet needs for optimizing care for older adults with HIV. Included studies are focused on two thematic areas. The first encompasses outcomes across the lifespan, including among younger and older PWH, and their comorbidities, mental health and the UNAIDS 90‐90‐90 targets for global HIV control (90% of PWH knowing their HIV status, 90% on ART and 90% virally suppressed) [5]. The second thematic area focuses on studies examining priorities for and approaches to improving how health systems care for and support PWH as they age.

Young adults with perinatally acquired HIV (YAPHIV) face unique challenges as they grow older with HIV. They are at increased risk of mental health comorbidities, such as depression, anxiety and substance use, as they transit to adulthood. This transition coincides with increased self‐awareness, establishing an identity and doing so within the setting of HIV‐associated stigma. Mental health issues can precipitate suicidal ideation, as has been reported among youth with other chronic illnesses [6, 7].

Kreniske et al. examine attempted suicide among YAPHIV and those perinatally exposed to but not living with HIV in the Child and Adolescent Self‐Awareness and Health study in New York City [8]. They studied the unique roles of socio‐demographic, contextual and psychosocial factors on mental health risks, such as how the impacts of pregnancy, substance use and HIV stigma differ. Worryingly, a quarter of YAPHIV in their study reported ever attempting suicide. Suicide attempts were associated with disorders of mood, anxiety and behaviour. They also were associated with higher occurrence of HIV stigma and pregnancy. These findings highlight the need to evaluate for suicidal risk among YAPHIV in care and of efforts to understand the magnitude and associated factors of all mental health challenges in relation to their peers.

Sirois et al. assessed the achievement of key early life milestones by YAPHIV in the US Perinatal HIV/AIDS Cohort national cohort [9]. Previous studies have suggested that YAPHIV have increased the risk of abnormal neurological development [10, 11, 12]. Their work expands on prior studies by focusing on evidence of achievement by YAPHIV, including high school diploma or graduate equivalency degree, postsecondary education, or employment, as well as factors associated with these milestones. While compelling, these potentially reassuring results warrant additional evaluations in different contexts to confirm outcomes and better understand other influences.

This supplement includes studies on renal disease, diabetes, mental health and sarcopenia to better understand comorbidities among ageing adult PWH. While questions abound regarding the relative impact of HIV and inflammation; ART medication, including drug–drug interactions and polypharmacy; behavioural factors, such as diet, smoking, substance use and physical activity; and environmental or genetic factors; it is clear that multiple causes contribute to the higher rates of comorbidities among PWH compared to those without HIV [13, 14, 15, 16, 17, 18].

Studying participants with and without HIV in the African Cohort Study, Chang et al. examined the prevalence and risk factors of renal insufficiency, elevated blood pressure, diabetes and dysglycemia [19]. Overall, older PWH were at higher risk for the comorbidities studied compared to PWH <50 years. Interestingly, while the overall prevalence of the comorbidities studied was high, it was not associated with HIV status. This is one of few studies from sub‐Saharan Africa comparing comorbidity risk between older PWH and community‐dwelling individuals without HIV. This study also raises questions regarding differences in the populations of those with and without HIV, and the need for better characterization of behavioural risks related to diet, physical activity, smoking and substance use.

Aurpibul et al. evaluated the prevalence and determinants of neurocognitive impairment (NCI) specifically amnestic mild cognitive impairment and dementia among older PWH from Northern Thailand who had a long history of HIV and ART [20]. The overall burden of NCI was substantial (87%), with 20% meeting their definition of dementia. While the prevalence of NCI in this study might be higher than has sometimes been seen in other studies [21], this raises questions regarding whether these differences are due to the population, the measures used or other factors.

Other co‐morbidity studies also demonstrate the need for more detailed and comparable research across social and cultural contexts. Luk et al. compared definitions of sarcopenia from the Asia Working Group of Sarcopenia, including consistency between criteria and construct validity as measured by associations with mobility and physical functioning among older PWH in Hong Kong [22]. This study is an example of the use of contextually applicable definitions to advance the field. Mwangala et al. present their study on the prevalence and predictors of symptoms for depression and generalized anxiety disorder among older PWH and those without HIV from Kenya with psychosocial factors, such as HIV stigma, household HIV burden and loneliness, all serving as risk indicators among PWH [23]. These findings reflect the value of longitudinal assessments of cognitive, physical and mental health to understand their impact on the trajectory of age‐associated decline among PWH.

These four studies on specific comorbidities among older PWH provide updates on the magnitude and burden of multimorbidity in the current ART era, including associated factors and modes of assessment in understudied regions. While higher prevalence rates for comorbidities were found in some but not all studies, risk factors differed among those with and without HIV, emphasizing the importance of considering comorbidities individually and careful evaluation among PWH to best improve clinical care and outcomes.

Farley et al. [24] provide a contemporaneous evaluation of the achievement of the UNAIDS 90‐90‐90 targets among older versus younger PWH in 13 African countries [5, 24]. In comparison to younger PWH, older PWH have previously been shown to have suboptimal HIV care and treatment outcomes, especially with regard to immunological and virological responses [25, 26]. Using representative Population‐based HIV Impact Assessments data, they found that older PWH achieved the second and third 90 (i.e. initiate ART and are virally suppressed). However, the first 90 on awareness of HIV status remains a challenge. Only 80% of older PWH in the study were aware of their HIV status, emphasizing the need for targeted interventions to enhance HIV prevention and testing in this population.

The pace of health transformation in low‐ and middle‐income countries (LMICs) most affected by the HIV epidemic needs to increase to provide chronic care for more PWH who are surviving longer and growing older. The article by Godfrey et al. describes how US PEPFAR‐supported countries now have the largest absolute numbers of older PWH, larger still than all of the older PWH in North America and Western Europe, combined [27]. This burden will only continue to increase in the coming years until an HIV cure is achieved. It is clear that innovative and differentiated service delivery (DSD) models will need to be employed in order to meet the non‐HIV health needs of older PWH in these resource‐constrained settings. Godfrey et al. outline lessons learned from PEPFAR programmes and suggest adaptations that may be considered. They argue that older PWH generally achieve good HIV treatment outcomes with high rates of adherence and viral suppression, but instead have other specific unmet needs, which include depression, cognitive impairment and frailty. PEPFAR‐supported HIV programmes have promoted implementing “simplified algorithms/therapies, task‐shifting and decentralize,” and suggest these principles to guide the development of DSD for older PWH. These models should incorporate the full extent of care from maximizing opportunities for diagnosis with locally validated tools to securing drug procurement and provision pipelines, as well as strengthening existing monitoring and evaluation. LMICs may leverage PEPFAR‐developed capacity‐building frameworks established to facilitate task‐shifting of HIV treatment and extend this to the management of other chronic comorbidities.

Linkage of health systems to the broader community for social support will be imperative for any model of care for an ageing population, especially given the increased reliance on formal support networks among older PWH. In their papers, Murzin et al. [28] and Reynolds et al. [29] use qualitative methods to better understand the health needs of older PWH in marginalized and rural communities from two diverse economies: Canada and Uganda. Both studies highlighted several similarities with that of the ageing non‐HIV population, drawing attention to the common, dominant role poor social and structural determinants of health play in influencing health and wellbeing. In many high‐income settings like Canada, support services may be available but poorly accessible by older PWH due to various reasons, as opposed to a complete lack of support services in low‐resource settings. Multiple needs—such as maintaining adequate nutrition and housing, mobility and sensory aids, in‐home support for daily activities and social/emotional support—were common in both settings and among those ageing without HIV. These studies also highlight how the lived experiences of PWH, including trauma and stigma, play a significant role in contributing to uncertainties about the future, which is an additional source of anxiety for ageing PWH.

Central to the transformation of any health system are the data informing its evolution and adaptation. Marty et al. utilized French national data to show that almost 20% of PWH will be >70 years of age by 2030, and 40% would have been on ART for >30 years [30]. By leveraging multiple research and surveillance resources, their team has provided evidence to guide local health systems to prepare for the levels of ageing‐related care these PWH will require. Their findings alert other contextually similar countries to the growing magnitude of older PWH in their communities.

The papers in this supplement emphasize the need to increase the capacity to design, test and evaluate different models of care for older PWH. This will be critical to inform our understanding of what works in different settings. Moreover, older PWH should be part of the teams designing and evaluating implementation strategies as their lived experiences are an invaluable resource for programmes. Service outcomes should include stigma‐reduction indicators and patient‐reported outcomes in addition to HIV‐related parameters as optimal health outcomes cannot be sustained over the long term without an integral plan to address stigma and quality of life. In addition, research should be dedicated to rigorous language and cultural adaptation and validation of critical tools needed to screen PWH for age‐associated and geriatric conditions, such as frailty and cognitive impairment. This is particularly critical given that many of the tools in current use were developed for the general elderly population (65+ years) in high‐income settings, and may not be well suited for younger PWH (50+ years) experiencing overlapping pathologies related to general ageing and HIV, nor for those in LMICs where there are far fewer screening and referral services.

In this supplement, studies show how research can be used to guide policy, care and treatment of individuals growing older with HIV. Health systems providing care for PWH need to evolve in response to the available evidence to meet the increasing health needs of both YAPHIV and older PWH beyond that of viral suppression. In many settings, HIV providers are the primary, if not the sole healthcare team overseeing the overall health of PWH, a situation that will invariably extend to managing non‐communicable diseases, and social and long‐term care as PWH age [31]. Countries will need to adopt integrated care approaches to HIV service provision that can be scalable in countries with a high HIV burden in order to achieve longer and richer healthspans for all those growing older with HIV.

## COMPETING INTERESTS

HMC has received funding for her institution from ViiV Healthcare. The other authors report no competing interests.

## AUTHORS’ CONTRIBUTIONS

All co‐authors drafted and revised the editorial, and approved the final version.

## FUNDING

Authors received funding from the US National Institutes of Health (R33AG067069, U01A1069911, UH3CA202723, U54CA254571), Agency for Healthcare Research and Quality's (AHRQ) and ViiV Health Care.

## DISCLAIMER

The views expressed in this article are the personal views of the author(s) and may not be understood or quoted as being made on behalf of or reflecting the position of the regulatory agency/agencies or organizations with which the author(s) is/are employed/affiliated.
